# The complete mitochondrial genome of *Plagiopholis styani* (Reptilia: Squamata: Colubridae)

**DOI:** 10.1080/23802359.2021.1962761

**Published:** 2021-09-27

**Authors:** Changkun Fu, Hailin Fan, Tianmeng Zhao, Hao Zong

**Affiliations:** College of Life Sciences, Sichuan Normal University, Chengdu, China

**Keywords:** Mitochondrial genome, phylogenetic tree, *Plagiopholis styani*, Colubridae

## Abstract

In this study, the complete mitochondrial genome of *Plagiopholis styani* was acquired and described. The mitogenome is a circular molecule of 19,669 base pairs (bp) in length including 13 protein-coding genes (PCGs), 22 transfer RNA (tRNA) genes, two ribosomal RNA (rRNA) genes, and two control regions (D-loop), which is similar to other Colubridae snakes. The total base composition of mitochondrial DNA is A 33.0%, C 24.9%, G 12.8%, and T 29.3%. The mitochondrial genome of *Plagiopholis styani* contributes to revealing the phylogenetic relationships among species of the Colubridae family.

*Plagiopholis styani* (Boulenger, 1899) belongs to the family Colubridae, and lives in the mountain area of 700–1000 m above sea level (Zhao 2003). The species is distributed in Sichuan, Fujian, Gansu Guangxi, Anhui, Zhejiang, Jiangxi provinces in China (Ji 2019). In this study, we sequenced the complete mitochondrial genome sequences of *P. styani* and combined with the existing mitochondrial genome sequences of Colubridae family in GenBank to construct a phylogenetic tree. These results can reveal the phylogenetic relationship between *P. styani* and other species in the Colubridae family.

The specimen of *P. styani* was collected from Mount Emei (Latitude: 29°34′20.01″N, Longitude: 103°23′41.63″E, Altitude: 751 m), and stored in the Zoological Museum (specimen number: EM1906004), Collage of Life Sciences, Sichuan Normal University, China. The email of the sample/DNA collector is fckgood@sina.com. The complete mitochondrial genome sequence was obtained by high-throughput sequencing method with Illumina Hiseq 2500 (Tsingke, Tianjin, China). The complete sequence of the mitochondrial genome was submitted to the GenBank.

The length of the mitochondrial genome of *P. styani* is 19,669 base pairs (bp) (GenBank accession number: MW697084), which contains 13 protein-coding genes (PCGs) (ATP6, ATP8, ND1, ND2, ND3, ND4, ND4L, ND5, ND6, COI, COII, COIII, Cytb), 22 transfer RNA (tRNA) genes, two ribosomal RNA (rRNA) genes, and two control regions (D-loop). The base composition is 33.0% for A, 29.3% for T, 24.9% for C, and 12.8% for G. The large ribosomal RNA (lrRNA) is 1468 bp in length and the small ribosomal RNA (srRNA) is 916 bp in length. The length of control area is 1488 bp. ND6 and seven tRNAs are encoded by the L-strand, whereas all the other genes are encoded by the H-strand.

Based on the concatenated nucleotide sequences of PCGs and two rRNAs, the phylogenetic relationships of the *P. styani* and the other 12 snakes were constructed by MEGA6.0 using maximum-likelihood (ML) method with 1000 bootstrap replications (Tamura et al. 2013). In the construction of the phylogenetic tree (Figure 1), the *Naja atra* was used as the outgroup. The phylogenetic tree showed that the genus *Plagiopholis* which *P. styani* belongs to was closer to the genus *Plagiopholis* in genetic relationship than others. The results of molecular phylogeny based on the mitochondrial genome are consistent with traditional morphological classification (Zhao 2003). This study provides data for the systematic classification of Colubridae. However, the molecular evidence inferred in this study is limited, more mitochondrial genomic information of other snakes is necessary to elucidate the evolutionary relationships within major lineages of Colubridae.

**Figure 1.**
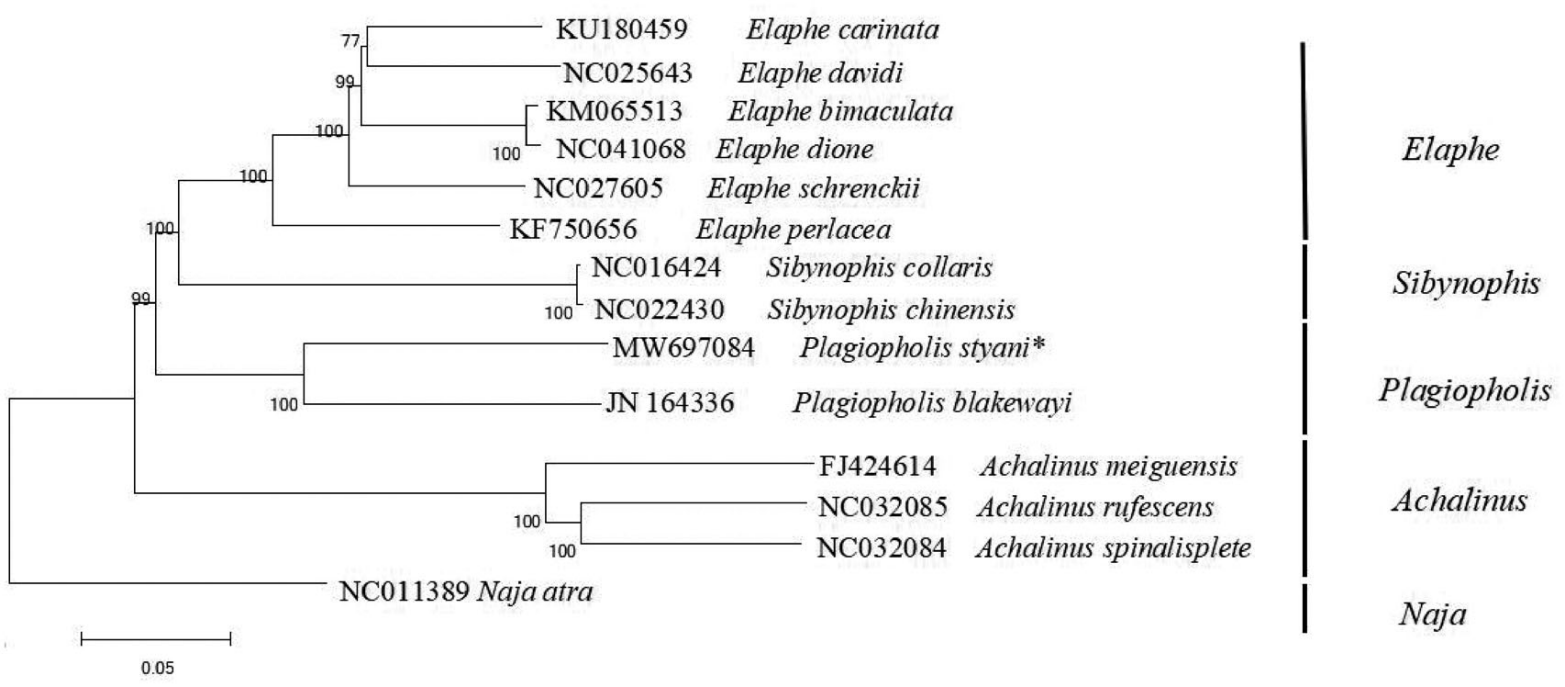
Phylogenetic tree inferred from maximum-likelihood analysis of the nucleotide of protein-coding genes and two ribosomal RNA genes. *Naja atra* was used as outgroup. The nodal numbers indicate the bootstrap values obtained with 1000 replicates. The GenBank accession number, species name, and generic name are shown on the right side of the phylogenetic tree. The newly sequenced mitogenome is indicated by the asterisk.

## Data Availability

The genome sequence data that support the findings of this study are openly available in GenBank of NCBI at https://www.ncbi.nlm.nih.gov/ under the accession no. MW697084. The associated BioProject, SRA, and Bio-Sample numbers are PRJNA706113, SRR13861122, and SAMN18117826, respectively.

## References

[CIT0001] Huang X, Yang DD, Zhang L, Zhang BW. 2014. Mitochondrial genome of *Protobothrops mangshanensis* (Squamata: Viperidae: Crotalinae). Mitochondrial DNA. 25(6):435–436. 23808922 10.3109/19401736.2013.809445

[CIT0002] Jang KH, Hwang UW. 2011. Complete mitochondrial genome of the black-headed snake *Sibynophis collaris* (Squamata, Serpentes, Colubridae). Mitochondrial DNA. 22(4):77–79. 22040070 10.3109/19401736.2011.624601

[CIT0003] Ji DM, Wen SS. 2002. Atlas of the reptiles of China. Zhengzhou, China: Henan Science and Technology Press.

[CIT0004] Ji LQ. 2019. China checklist of animals. Catalogue of life China: 2019 annual checklist. Beijing, China: The Biodiversity Committee of Chinese Academy of Sciences.

[CIT0005] Tamura K, Stecher G, Peterson D, Filipski A, Kumar S. 2013. MEGA6: molecular evolutionary genetics analysis version 6.0. Mol Biol Evol. 30(12):2725–2729. 24132122 10.1093/molbev/mst197 PMC3840312

[CIT0006] Wolstenholme DR. 1992. Animal mitochondrial DNA: structure and evolution. Int Rev Cytol. 141(6):173–216. 1452431 10.1016/s0074-7696(08)62066-5

[CIT0007] Woo H-J, Ryu SH, Jang KH, Choi EH, Kim SK, Hwang UW. 2009. Mitochondrial genome of the Korean colubrid snake *Elaphe schrenckii* (Reptilia; Squamata; Colubridae). Mitochondrial DNA. 20(5–6):107–109. 19900059 10.3109/19401730903137088

[CIT0008] Zhang L, Huang X, Han D, Xue C, Zhang B. 2015. Mitochondrial genome of *Protobothrops cornutus* (Squamata: Viperidae: Crotalinae). Mitochondrial DNA. 26(2):278–279. 24021010 10.3109/19401736.2013.825774

[CIT0009] Zhao EM. 2003. Coloured atlas of Sichuan reptiles. Beijing, China: China Forestry Publishing House.

